# Review on the Application of Low-Field Nuclear Magnetic Resonance Technology in Coalbed Methane Production Simulation

**DOI:** 10.1021/acsomega.2c02112

**Published:** 2022-07-20

**Authors:** Junjian Zhang, Xuanxuan Chu, Chongtao Wei, Pengfei Zhang, Mingjun Zou, Boyang Wang, Fangkai Quan, Wei Ju

**Affiliations:** † College of Earth Sciences & Engineering, Shandong University of Science and Technology, Qingdao 266590, China; ‡ School of Resources and Earth Science, China University of Mining and Technology, Xuzhou 221116, China; § Department of Civil Engineering, University of Nottingham, Nottingham NG7 2RD, U.K.; ∥ College of Geosciences and Engineering, North China University of Water Resources and Electric Power, Zhengzhou 450045, China; ⊥ Key Laboratory of Continental Shale Hydrocarbon Accumulation and Efficient Development, Ministry of Education, Northeast Petroleum University, Daqing 163318,China

## Abstract

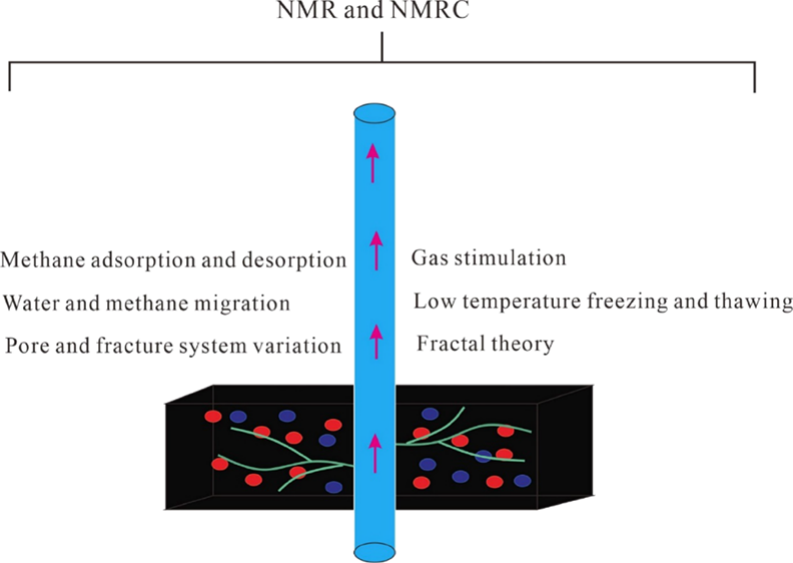

Low-field nuclear magnetic resonance has become one of the main methods to characterize static parameters and dynamic changes in unconventional reservoirs. The research focus of this paper is process simulation of coalbed methane (CBM) production. The dynamic variation of pore volume with different pore sizes during pressure drop, methane desorption–diffusion process, and methane–water interaction during migration is discussed. Moreover, the calculation principles of NMR single and multifractal models are systematically described, and the applicability of NMR fractal models within different research contexts is discussed. Four aspects need urgent attention in the application of this technology in CBM production: (1) overburden NMR technology has limitations in characterizing the stress sensitivity of shale and high-rank coal reservoirs with micropores developed, and we should aim to enable an accurate description of micropore pore stress sensitivity; (2) dynamic NMR physical simulation of reservoir gas and water production based on in-situ and actual geological development conditions should become one of the key aspects of follow-up research; (3) low-temperature freeze–thaw NMR technology, as a new pore–fracture characterization method, needs to be further applied in characterizing the distribution characteristics of pores and fractures; and (4) NMR fractal model should be used as the main theoretical method to expand the simulation results. The applicability of different fractal models in characterizing pore–fracture structure (static) and CBM production process (dynamic) needs to be clarified.

## 1 Introduction

Coalbed methane (CBM) production includes adsorbed methane desorption on the surface of coal matrix during drainage and depressurization, methane transport from the matrix to the fracture by diffusion, and further transport to the wellbore through fracture by Darcy seepage. Therefore, the desorption–diffusion–seepage process of methane and water has been the core link through CBM production.^1,2^ The fine characterization of pore–fracture structural evolution, methane adsorption–desorption, and gas–water transport variation in coal reservoirs by experimental methods has become a prerequisite for an in-depth understanding of CBM production. The low-field nuclear magnetic resonance (LF-NMR) technique has the advantages of no disturbance to samples, accurate detection, and high resolution and can quantitatively identify the hydrogen nuclei content in water. It has been widely used to study pore–fracture structure characterization, morphology, size, and porosity.^3−5^ Subsequently, many scholars focus on methane and propose a calibration method of quantitatively characterizing methane content of different phases using NMR. This provides an idea for studying real-time methane desorption–diffusion during CBM extraction.^6^ Based on the abovementioned theory, many scholars have carried out relevant studies using NMR technology from the perspectives of gas competitive adsorption, gas–water transport, and gas injection stimulation and have achieved progress.

However, it is worth noting that as unconventional reservoirs, coal reservoirs are characterized by poor porosity, poor permeability, and stronger heterogeneity. There are clear differences in coal reservoirs in different coal-bearing basins. The comparison of related literature shows that the stress sensitivity of coal samples with different coal ranks (maturity)and the stress sensitivity of pores with different pore sizes in the same coal sample are different. This variability is mostly reported in the literature of NMR simulations related to methane desorption and gas–water transport due to the physical characteristics of the coal rock mass and the debatable applicability of the NMR technique to the CBM simulation. Thus, this paper presents the research method and the understanding of NMR technology in characterizing the pore–fracture structure evolution, gas–water transport, and gas injection stimulation during the pressure drop process. In addition, we focus on discrepant conclusions in NMR-based studies and discuss these. The key research directions and challenges in terms of NMR application to CBM production are also proposed.

## 2 Results and Discussion

### 2.1 Dynamic Simulation of CBM Production

LF-NMR theory mainly includes two aspects, that is, pore classification using the one-dimensional *T*_2_ spectrum and fluid identification using the two-dimensional *T*_1_–*T*_2_ spectrum. The former is used to characterize pore and fracture structures and micro-occurrence of a single fluid. The latter is used to compare the difference of multifluid occurrence. A large number of studies have described the two technical principles in detail.^7−9^ This paper mainly discusses the specific application and related problems of the LF-NMR technology in the CBM production process.

#### 2.1.1 Dynamic Variation of Reservoirs’ Pore–Fracture Structure

**Figure 1:**
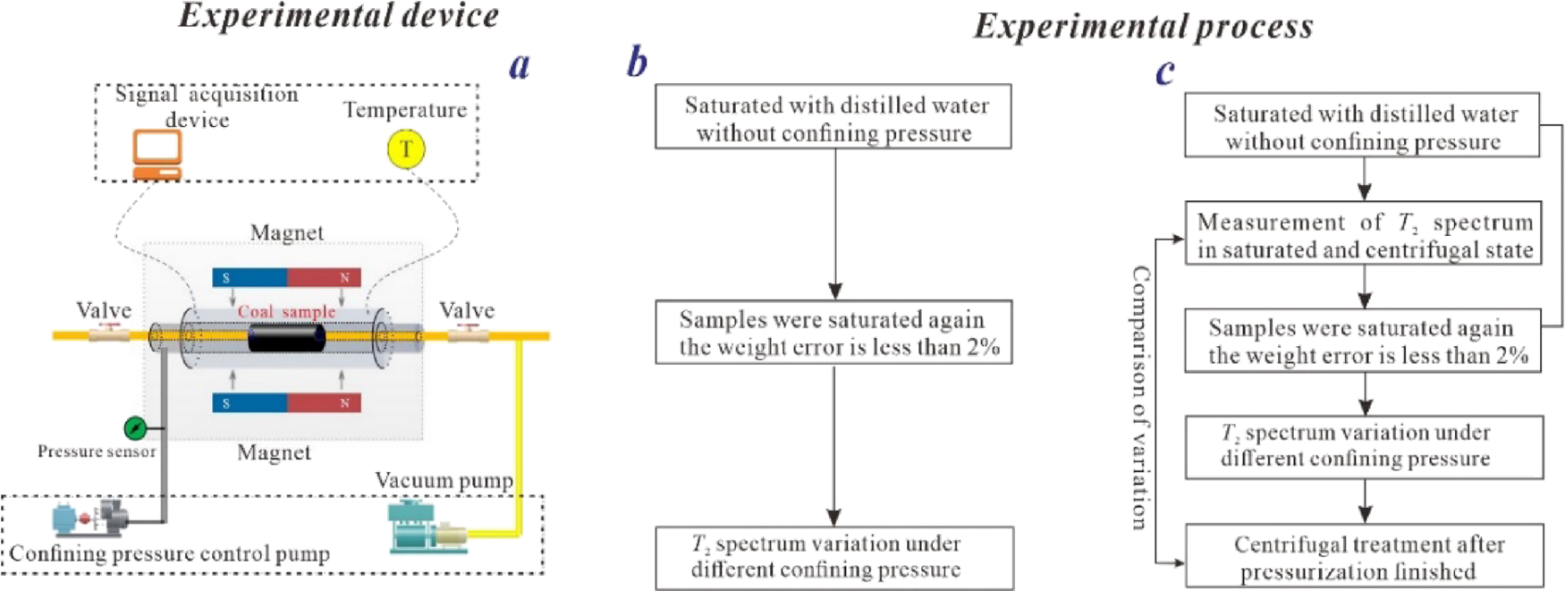
Experimental setup and flowchart of stress sensitivity tests of saturated coal samples. (a) NMR experimental device; (b) *T*_2_ spectrum variation under the effect of confining pressure; (c) bound water variation of *T*_2_ spectrum variation under the effect of confining pressure).

Before the pressure drops to the critical desorption pressure of methane in coal reservoirs during CBM drainage, the increase of effective stresses is the dominant factor leading to pore–fracture structure variation of coal reservoirs.^10−13^ To separately describe the stress sensitivity of pores with different diameters under the effective stress, Li et al.^11^ initially used NMR to obtain the *T*_2_ spectrum distribution of low-, medium-, and high-rank saturated coal samples (25 × 50 mm cylindrical samples) by increasing the confining pressure; the volumetric compressibility coefficients of adsorption pore, seepage pore, and fracture were calculated; a volumetric compressibility coefficient model using LF-NMR tests was constructed. The results show that the compressible space of adsorption pores in the same coal sample was significantly lower than that of seepage pores and fractures, and the corresponding pore volume varied exponentially with increasing stresses. Thus, most scholars have explored the variation characteristics of pore–fracture *T*_2_ spectra with confining stress using different coal ranks following the procedure shown in Figure 1a,b, and the factors affecting stress sensitivity were investigated from maceral and mineral composition.^14−17^

**Figure 2:**
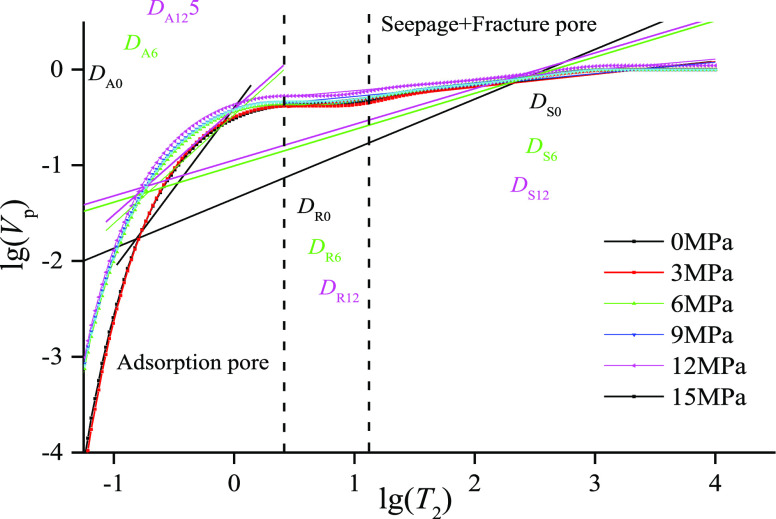
Fractal calculation by using variation of *T*_2_ spectra at different confining pressures.

In order to make further advances in this research, based on the *T*_2_ spectra distribution curves under different confining pressures, Zhang et al.^13^ and Chen et al.^16^ calculated the fractal dimension values of adsorption pores, seepage pores, and fractures at different stresses (*D*_ad_, *D*_se/fracture_, and *D*_total_) using the NMR single fractal model; the effect of stress on the pore size distribution heterogeneity with different diameters was quantitatively characterized (Figure 2). The correlation of fractal parameters with compressibility coefficients as well as the correlation of permeability and adsorption constants were analyzed to explore the relationship between stress sensitivity and heterogeneity dynamic variation in coal samples. The NMR fractal model applicability is presented in Section 3.

**Figure 3:**
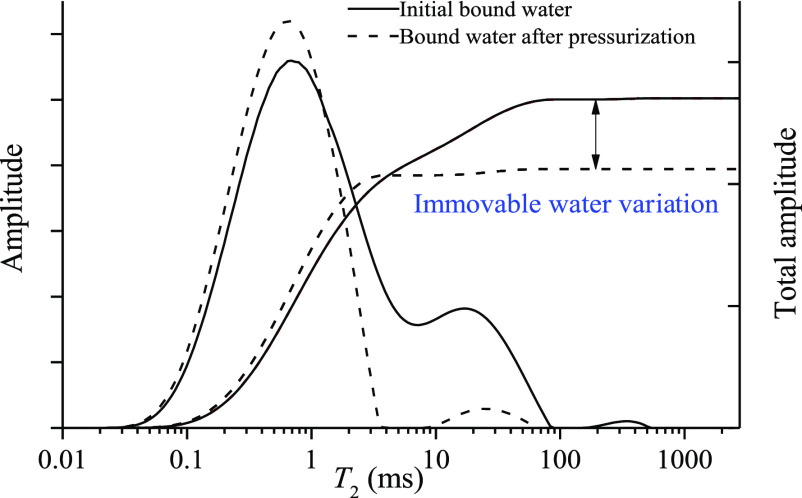
*T*_2_ spectrum distribution of bound water in coal samples before and after applying stress.

Then, based on overburden NMR tests and the *T*_2_ spectrum curves obtained after centrifugation, Zhang et al.^18^ and Hou et al.^19,20^ explored the stress sensitivity of bound water saturation in different coal-rank reservoirs by comparing the differences in the *T*_2_ spectrum distribution of bound water in coal samples under different stresses (Figure 1c). The results show that the bound water saturation of medium- and high-rank coal samples exhibited significant stress sensitivity. The distribution state of bound water in coal reservoirs and its transport and conversion under stress has received increasing attention from scholars, and the application of NMR technology in this field should be further emphasized (Figures 2 and 3).

Although the overburden NMR technique has become an emerging method of characterizing the stress sensitivity of coal reservoirs, the following problems remain unsolved. (1) The *T*_2_ adsorption pore spectrum area of some low-rank coal samples gradually increases as the confining stress increases, indicating that the micropore volume gradually increases with the increase of pressure.^16,20^ It is suggested that the seepage pores and fractures in the coal samples are relatively developed. The compressible volumes of both are large under pressure and some of the larger pores are converted into adsorption pores, resulting in the gradual increase of the micropore volume. Thus, the applicability of the overburden NMR technique to analyze the pore stress sensitivity with different diameters is debatable, and the overall compressibility coefficient should be used to characterize the stress sensitivity of coal samples. (2) For the high-rank coal samples with underdeveloped fractures, the *T*_2_ spectral peaks of adsorption pores are developed at the initial conditions, and the adsorption pores provide the main compressible space. However, the *T*_2_ spectral area variation under stress is small and the area variation rate does not exceed 10%, resulting in a lower calculation accuracy of adsorption pore compressible space and thus affecting the accuracy of analysis results.^16,20^ In summary, the technique still has limitations in characterizing stress sensitivity in unconventional reservoirs, which also restricts the study of the influence of stress sensitivity on the permeability of the CBM drainage process.

#### 2.1.2 Methane Desorption–Diffusion Processes in Different Phases

When the reservoir pressure decreases to the critical methane desorption pressure, desorption of adsorbed methane on the coal matrix surface occurs and the coal matrix shrinks, leading to a gradual increase in reservoir porosity and permeability. The effective stress and matrix shrinkage effect is the core factors affecting the coal pore–fracture structure.^21−23^ In this stage, the LF-NMR technique is mostly used to simulate the methane desorption process and has the core advantage of monitoring the conversion and transport process of methane in different phase states in real time. Tang et al.^23^ used the NMR technique to simulate the natural desorption process of coal samples, and the results show that the adsorption and desorption curves of dry coal samples varied logarithmically with time, while the moisture content had a significant control on the adsorption–desorption amount. However, the gas production during CBM extraction is a stepwise pressure reduction process, and the methane desorption process in the aforementioned literature is atmospheric desorption at atmospheric outlet pressure (0.1 MPa), which does not match the actual discharge process. Thus, by using this technique, Zhang et al.^24^ and Quan et al.^25^ realized the simulation of stepwise depressurization desorption of methane from medium- and high-rank coal samples in the Eastern Yunnan area by adjusting the non-return valve of the high-temperature and high-pressure repulsion NMR device and decreasing the outlet pressure in a stepwise manner.

Results show that methane desorption rate with stepwise pressure drop was significantly higher than that of natural desorption, and there were clear differences in methane desorption at different pressure drop gradients. To mechanistically explain the effect of the pressure drop rate on methane desorption and transport process, Quan et al.^25^ established a methane diffusion coefficient calculation model using NMR test results and a classical model of methane diffusion coefficients. The calculation results show that the increase of effective diffusion coefficients at a certain pressure drop rate induced higher desorption efficiency by stepwise pressure drop than by natural desorption. In addition, Li et al.^26^ investigated the methane adsorption under stepwise pressure drop using the NMR technique and calculated the micropore diffusion coefficient based on the bidisperse model and the dynamic diffusion coefficient based on the multiporous model for the adsorption process, respectively. The results show that their diffusion coefficients showed consistent variations, and the dynamic variation of diffusion coefficients was related to the combined effect of methane diffusion mechanism and coal matrix swelling under different adsorption pressures.

In summary, most of the studies in this area focus on the adsorbed/free methane transport under atmospheric (variable) pressure conditions, with emphasis on the interconversion of different phases of methane at different pressure drop rates. By counting the adsorbed/free methane content at different times and calculating the diffusion coefficients through the classical diffusion model, the influencing mechanism of the pressure drop rate on the methane desorption and diffusion process is elaborated from a microscopic perspective. Currently, this method can be used to study multiphase methane transport in the CBM drainage process in the coal reservoir development area with different coal ranks, tectonic deformation degrees, and water-bearing conditions. In addition, it is worth noting that the NMR technique is mainly used to describe the methane desorption–diffusion process at different initial conditions (e.g., pressure drop rates and water saturation). The pore–fracture structure evolution is the key to explaining this microscopic transport process. Exploring the variation of the pore–fracture structure with different pore diameters should be closely integrated with Section 2.1.

#### 2.1.3 Gas and Water Two-Phase Fluid Transport

As the methane drainage continues, the water saturation of the coal reservoir gradually decreases, and the gas–water interaction becomes an important link to restrict CBM production. The microscopic transport law of water in coal reservoirs is the key to an accurate understanding of this issue. Water injection simulation studies based on LF-NMR technology are relatively mature, and the available results show that the ease of water entry into pores is inversely proportional to the pore size, that is, water preferentially enters the macropores, followed by mesopores and micropores.^24^ Thus, it is mostly used to discuss the replacement mechanisms such as water-driven gas and gas-driven water. Based on a comprehensive analysis of current studies, the authors think that there is a significant difference in the influencing mechanism of water on the adsorbed/free methane replacement. First, water molecules enter the coal nanopores (adsorption pores) at a specific injection pressure and flow by attaching to the pore surface due to the preferential flow effect in the overburden mode, thus replacing the adsorbed methane from the nanopore surface. However, for semi-open pores, which are open at one end and closed at the other end, an increase in water injection pressure may lead to an increase in pressure inside the pore. This may cause the free methane in the pore to reattach to the pore surface. Second, water drives free methane in macropores or fractures mainly through applied pressure. It is essentially a gradual filling of the pore space by water. The free methane is mainly replaced outside the coal sample in the form of volume replacement. With the increase of the repelling time, the injection volume gradually increases, and more free methane is replaced.^27^

**Figure 4:**
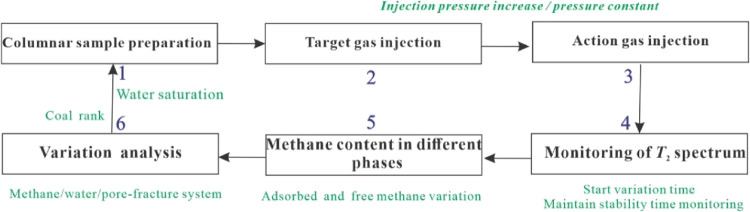
Flowchart of gas replacement tests.

To enhance the CBM production, a series of injection stimulation techniques have been introduced. The nature of N_2_/CO_2_ injection stimulation techniques results from the competitive adsorption/desorption interactions of different gases. A large number of scholars have analyzed the interaction between methane and related gases in the adsorption/desorption process with N_2_-methane, CO_2_-methane, and water-methane as the research targets and generally agreed that N_2_/CO_2_ has good replacement effects. The general idea behind the mentioned studies is to analyze the *T*_2_ spectral wave volumes in the adsorbed and free methane to quantitatively characterize the dynamic methane transport processes. However, it should be noted that most of the gas replacement tests for unconventional reservoirs are unable to visualize the distribution characteristics of methane/water in the cross-section of column samples due to unavailable good LF-NMR 2D imaging. Due to the development of micropores in unconventional reservoirs, the sample cross-section has a low water content, resulting in poor imaging results. It should be focused on strengthening the research of the probe liquid medium and also improving the signal accuracy of the existing equipment. These measures are the key to solving this problem (Figure 4).

### 2.2 Application of Liquid N_2_/CO_2_ Freeze–Thaw Technology

#### 2.2.1 Changes in Reservoir Physical Properties under Freeze–Thaw Damage

Hydraulic fracturing production enhancement is one of the key means to improve CBM production capacity in low-permeability reservoirs, while this method has problems such as water lock effect, large water consumption, and environmental pollution of fracturing fluid. In contrast, cryogenic liquid freeze fracturing technology (i.e., fracturing coal by periodically injecting liquid N_2_, CO_2_, and other low-temperature fluids into the coal body), as a water-free fracturing method, can not only effectively avoid excessive waste of water, but also provide significantly strong injection and drainage effects. This technique has gradually received the attention of many scholars.^28−31^

During N_2_ (CO_2_) injection, the reservoir pore–fracture space shrinks or expands under the dual effect of temperature stress and freeze-heaving force, and the microscopic pore–fracture structure of coal reservoir changes, which affects the desorption–diffusion and seepage of methane.^28^ At present, numerous scholars have taken the physical properties of coal and pore–fracture structure changes under the effect of freeze–thaw damage of low-temperature liquids as the entry point to analyze the dynamic changes of permeability under the evolution of pore–fracture structure. The comparison of pore structures with different diameters before and after freeze–thawing using the LF-NMR technique has become the main method in this field.

Relative to the dynamic process of permeability, the amount of methane desorption and diffusion in coal reservoirs is key to obtain a breakthrough in CBM production capacity. The current research on the influence of low-temperature freeze–thaw on the dynamic process of methane desorption and production needs to be carried out in depth. In addition, as the water saturation of coal reservoirs gradually decreases with continuous drainage, the gas–water interaction in the reservoir also becomes one of the main factors affecting the low-temperature freeze–thaw effect. Currently, the study of the dynamic response mechanism of methane desorption and diffusion in water-bearing coal reservoirs during low-temperature freeze–thaw has been the focus in this field.

The comparison with related literature shows that the effects of water on methane adsorption, desorption, and gas–water permeability vary in different coal samples at the same water content (saturation). This can be attributed to the difference in the microscopic distribution of water in coal reservoirs caused by the non-homogeneity of the pore–fracture structure. Clarifying the bound/movable water (adsorbed/free water) content (saturation) in coal reservoirs is the basis for an accurate understanding of this problem. At present, many scholars have calculated the saturation of bound water in coal reservoirs using NMR saturation and centrifugal methods. The key factors affecting the bound water content of coal reservoirs have been discussed from the perspectives of burial conditions, coal rock basic components, and pore structure characteristics.

However, the abovementioned methods can only study the overall bound water content, and it is difficult to quantitatively characterize the multiphase water content such as bound water and free water at a specific water content (saturation). It is found that the two-dimensional (2D) NMR technique has high discriminating power in identifying different fluid components and multiscale nanopores compared to the conventional one-dimensional (1D) NMR technique. Therefore, this technique is gradually used for quantitative and fine characterization of different fluid components (e.g., bound water, free water, oil, and gas) in pores. Using this technique, Sun^32^ classified water in coal reservoirs into free water, bound water in inorganic pores, bound water in organic pores, and hydrogen-bearing material in the matrix and explored the content of each phase of water in coal samples with different coal ranks. At present, the technical difficulties mainly lie in obtaining high precision *T*_1_–*T*_2_ spectra, and the 2D spectral technique is an important method to comprehensively understand the low-temperature freeze–thaw fracturing theory.

#### 2.2.2 Characterization of NMRC Pore Structure under Freeze–Thaw Action

Scanning electron microscopy, mercury-pressure method, N_2_ adsorption method, and computerized tomography method have been extensively used to study the pore characteristics of shale. However, due to the complex and non-homogeneous pore structure of unconventional reservoirs (shale and coal), these methods are usually limited in terms of applicability and characterization testing accuracy. Apart from the mechanism of low-temperature liquid freeze–thaw damage, some scholars start to analyze the kinetic behavior of liquids at the nanoscale. Based on the Gibbs–Thomson equation and the total *T*_2_ spectra at different temperatures, the characteristics of nanoscale pore distribution can be achieved. This is collectively referred to as NMR cryoporosimetry (NMRC).^33,34^

**Figure 5:**
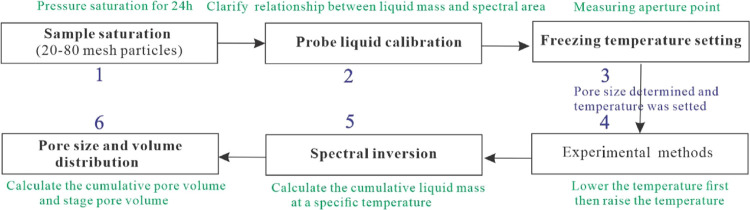
Flowchart of the low-temperature freeze–thaw NMR technique.

**Figure 6:**
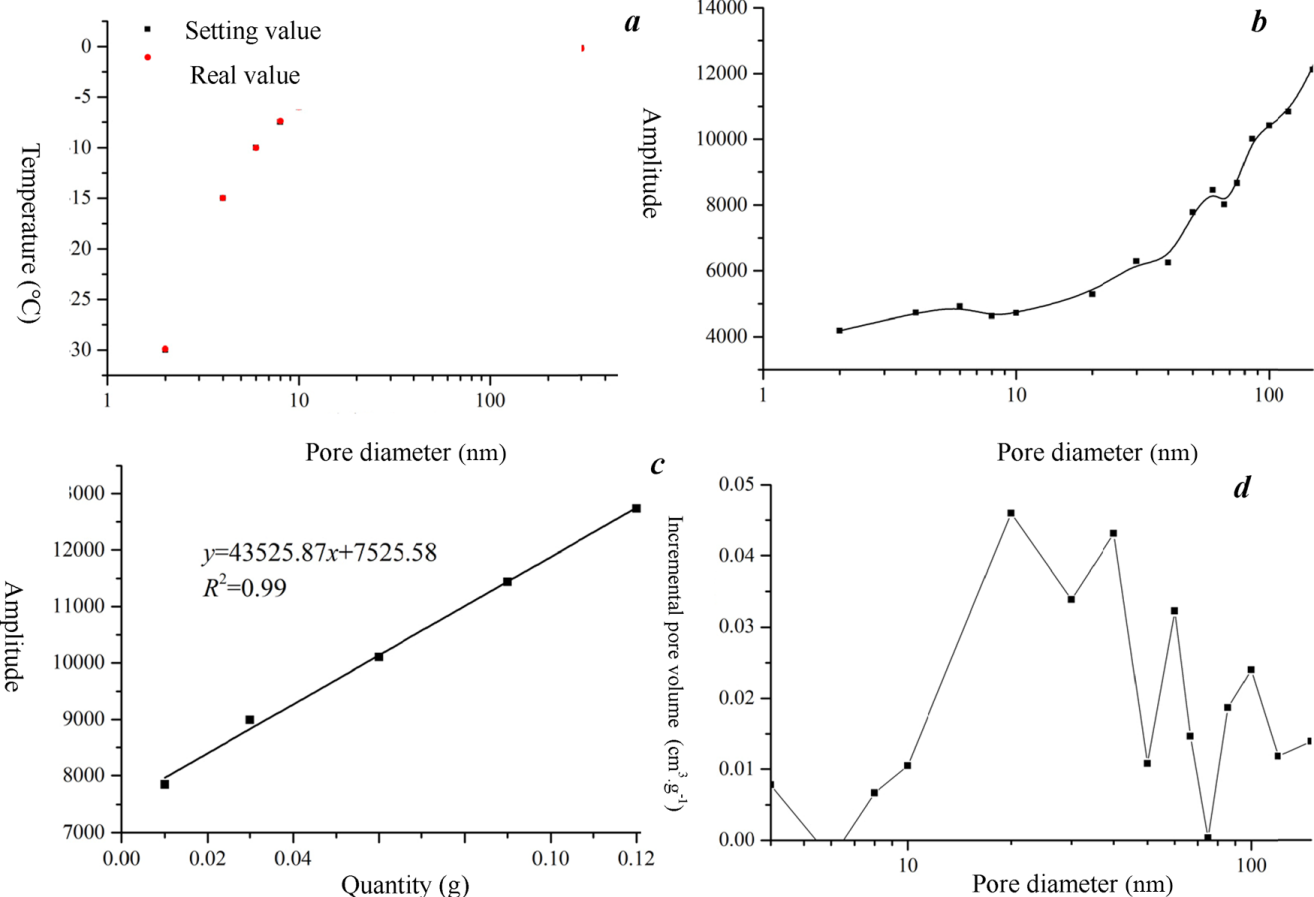
Characterization of TC15 pore distribution of coal samples based on the NMRC technique. (a) Temperature setting; (b) relationship between pore diameter and amplitude; (c) relationship between quantity and amplitude; (d) pore size distribution by using NMRC tests.

Liu^33^ and Zhang et al.^24^ performed NMRC tests using water as the probe liquid. After the liquid calibration of the medium-rank coal sample using the Newmark MesoMR12-070H-I integrated instrument with a low-temperature freeze–thaw instrument (Figure 5), NMRC tests were conducted. The temperature was set from −30 to 0 °C (Figure 6a), and the pore size ranged from 2 to 600 nm (Figure 6c,d). To compare the liquid N_2_ test data, the focus was on the pore distribution characteristics of pores with a diameter of 2–100 nm. Comparing the data of the same sample from Zhang et al.,^24^ the pore size distribution trend in each phase was quite consistent with the liquid N_2_ test results. However, it should be noted that the pore capacity obtained by this test method was one order of magnitude larger than that by low-temperature N_2_ adsorption, which may be related to the strong hydrophilicity of this coal sample within this pore range.

As an emerging method for pore testing, the NMR freeze–thaw method has gradually gained the attention of related scholars, but it is still in the initial stage and related problems still need to be solved. Liu et al.^33^ thought that sample size, probe liquid, freeze–thaw damage, and mineral content would all have an impact on the experimental accuracy. We think that the core problem for promoting this theory is to solve the pore structure changes under the influence of freeze–thaw damage (Section 2.1). A large amount of literature shows that freeze–thaw damage mostly occurs in macropores such as fractures, and the influence on the pore structure of small pores is relatively small. Thus, exploring the critical value of pore size affected by freeze–thaw damage is one of the urgent problems to solve. In addition, the applicability of particle size for testing different types of unconventional reservoirs also needs to be fully considered.

### 2.3 Applicability and Characterization Significance of the NMR Fractal Model

**Figure 7:**
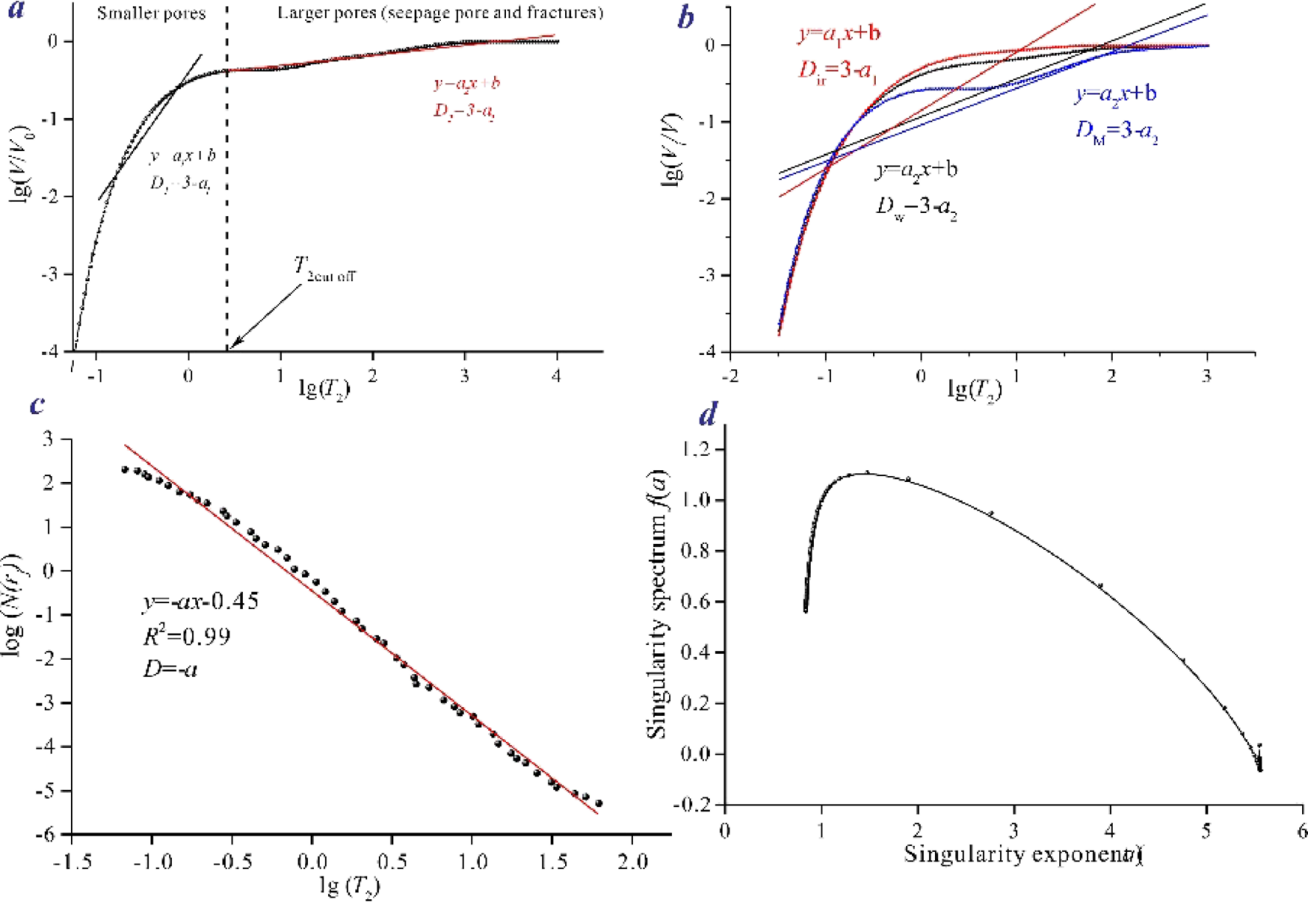
Calculation process of different fractal models. (a) Fractal curve by using model 1; (b) fractal curves by using model 2; (c) fractal curve by using model 3; and (d) fractal curve by using model 4.

The fractal theory has become one of the main methods to extend NMR simulation test results. The fractal models of NMR *T*_2_ spectra for unconventional reservoirs can be summarized as single fractal and multiple fractal models. Zhang and Hu^35^ studied dense sandstone reservoirs in Ordos Basin and explored the applicability of fractal models in characterizing the heterogeneity of pore–fracture distribution in reservoirs based on the systematic description of the calculation principles of each fractal model, the pore-permeability parameters, pore structure, and other factors (Figure 7) . However, the applicability of fractal models to other reservoir types such as coal reservoirs has not been reported. In addition, CBM production is a long-term dynamic process, and the NMR fractal values of the same coal sample are mostly calculated under different confining pressure, desorption pressure, and gas–water action. Compared with static characterization, dynamic characterization by the analytical models shows more difficulties in terms of applicability. It is of great theoretical and practical significance to solve the abovementioned problems in order to expand the NMR application and to gain a deep understanding of the mechanism of CBM production (Figure 7).

#### 2.3.1 Single Fractal Model

**Model 1**: This model is the most commonly used NMR fractal model, which is mainly used to characterize the structural features of pore–fracture morphology:^35^
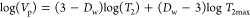
1where *V*_p_ is the cumulative pore volume (amplitude) percentage corresponding to the *T*_2_ spectrum in the saturated water state; *T*_2max_ is the maximum lateral relaxation time, ms; and *D*_w_ is the NMR fractal dimensional value in the water saturation.^13^

As shown in Figure 7a, the incompressible space and compressible space fractal dimensional values can be obtained using the *T*_2_ cutoff value. The adsorption pore, seepage pore, and fractal dimensional values can be obtained using the pore classification method.

**Model 2**. When the pore morphology of macropores is complex, the centrifugal force is difficult to overcome the capillary force, resulting in the occurrence of some bound water in macropores. Thus, some scholars think that the fractal value of bound water calculated by Model 1 may not accurately characterize the distribution of this part of water, while curves 2 and 3 can truly represent the distribution state of bound water and movable water in the reservoir pores. Based on eq 1, the fractal calculation was carried out using curves 2 and 3 in Figure 7a as the database, and the detailed calculation process is shown in Model 2 (Figure 7b). The results show that the total pore space of the sample (including movable and bound water) and the heterogeneous distribution of bound water in the pore space can be obtained through full-range linear fitting.^31^

**Model 3**. Lai et al.^36^ derived a fractal model on the NMR *T*_2_ spectra since dense sandstone pores can be quantitatively described as cylindrical and spherical pores.

For cylindrical pores,
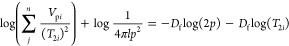
2

For spherical pores,
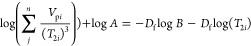
3where *r* is the pore diameter, nm; *N_i_* is the number of pores with pore diameter equal to *r_i_*; *D*_f_ is the pore fractal dimension, ranging between 2 and 3; *V*_p_ is the pore volume of pores with a specific diameter *r*.

Using eqs 2 and 3, the fractal values based on different pore morphology models can be obtained, respectively. The calculation process of the model is shown in Figure 7c (Model 3), and the results show that the model can achieve a quantitative characterization of the full-scale pore structure and has a good linear correlation with the pore structure parameters.

#### 2.3.2 Multifractal Model

For strongly inhomogeneous reservoirs, the pore size distribution curve usually fluctuates or jumps randomly and may exhibit different types of self-similarity at different pore size intervals. Thus, it is difficult to fully characterize the homogeneity of the pore space by a single fractal dimension.^37,38^ The calculation process of this model is shown in Figure 7d (Model 4), and the calculation process of multiple fractals and the physical characterization meaning of parameters are detailed in ref (39−42). It should be noted that the multifractal calculation results include two expressions, that is, *a* ∼ *f*(*a*) singular spectrum and *q* ∼ *D* fractal dimension spectrum. The values of these two expressions have a clear linear correlation, and thus we suggest that either expression can be selected for the multifractal calculation using NMR data. The description of both expressions in the same literature is essentially a meaningless duplication of research. By taking the singular spectrum parameters as an example, the characteristic parameters mainly include *a*_min_, *a*_max_, *a*_0_, *a*_0_-*a*_max_, *a*_min_-*a*_0_, and *A*. Particularly, the right branch width (*a*_min_-*a*_0_), the left branch (*a*_0_-*a*_max_), and *a*_min_-*a*_max_ characterize the distribution non-homogeneity of the data. For the NMR *T*_2_ spectra, larger *a*_min_-*a*_0_ indicates a more inhomogeneous pore size distribution in the low pore volume development area, while higher *a*_0_-*a*_max_ indicates a more inhomogeneous pore size distribution in the high pore volume development area.

#### 2.3.3 Applicability of Fractal Models

We retrieved the published literature to date through *Web of Science* using the keywords “fractal dimension” and “NMR”. The results show that most of the literature uses the four abovementioned fractal models to calculate the fractal dimension of the reservoir static parameters. However, from Figure 6 and Table 1, it can be seen that most of the literature uses eq 1 to explore the changes of adsorption/seepage/fracture structure during gas–water transport under stress. Combined with the statistical results, we consider that the following problems remain to be solved.

- Whether the fractal dimension value *D*_A_ of adsorption pores is characteristically significant remains to be analyzed. Some scholars think that the *D*_A_ value less than 2 does not satisfy the fractal range, and therefore the adsorption pores are not considered to have fractal characteristics.^13^ Other scholars think that this pore size range has fractal characteristics and can be used to calculate the fractal dimension value to analyze the non-homogeneous variation of pore distribution of adsorption pores.^10^ The coexistence of two seemingly contradictory conclusions has also affected the application and expansion of fractal models.
- The applicability of the fractal model to characterize the dynamic changes of pore–fracture inhomogeneity needs to be further validated. From Table 1, it can be seen that the differences in the fractal dimension values from the relevant literature are relatively small, with a minimum *D*_A_ variation of 0.032^27^ and a minimum *D*_S_ variation of 0.02.^13^ The linear fitting shows that a low coefficient of determination (*R*^2^) is mostly less than 0.6 when calculating bound water, movable water, and the total distribution inhomogeneity using Models 1 and 2 (Table 1). Both factors affect the accuracy of the research results. Particularly, smaller variations in the values of external factors (e.g., pressure and number of low-temperature cycles) result in a lower degree of modification of the pore–fracture structure in the coal reservoir. The difference between the fractal dimension values obtained using the two models is small, which can easily lead to data errors.
- The applicability of different NMR fractal models needs to be further verified. Based on the systematic elaboration of the principles of each fractal model, we suggest focusing on the introduction of Models 3 and 4 into the characterization of non-homogeneity of unconventional reservoir-dense sandstone in future studies. Compared with Models 1 and 2, Models 3 and 4 both show better linear relationships. The calculation accuracy of Model 3 is more influenced by the selection of the pore size interval.^31^ However, compared with dense sandstone reservoirs, coal reservoirs have a wider distribution of coal ranks. The NMR *T*_2_ spectra of low- and medium-rank coal show a clear triple-peak feature, whereas the *T*_2_ spectra of high-rank coal samples with undeveloped fractures show a clear single-peak feature. The differences in the pore–fracture distribution of different coal-rank samples inevitably reduce the applicability of different NMR fractal models. Therefore, in the dynamic characterization of the inhomogeneity of pore and fluid distribution in CBM production and transport, studies on the applicability of NMR fractal models should be enhanced.

**Table 1:** Selected Literature and Calculation Results of NMR Fractal Model Applications^a^

application	fractal models	*R*_o,max_ (%)	variable parameters	variation of fractal dimension values	*R*^2^	literature source
fracturing and gas injection stimulation	model 1	0.331 (low rank)	liquid N_2_ freezing time: 1–60 min	*D*_A_:1.375–1.343	0.84–0.87	Qin et al.^30^
				*D*_S_: 2.956–2.895	0.89–0.92	
	model 2		number of freezing cycles: 1–30	*D*_ir_: 2.58–2.597	0.45–0.46	
				*D*_F_: 2.33–2.071	0.62–0.69	
				*D*_T_: 2.408–2.313	0.62–0.67	
	model 1		supercritical carbon dioxide freezing: 1–13 d	*D*_A_: 1.5962–1.6070	0.74–0.74	Su et al.^42^
				*D*_S_: 2.9638–2.9710	0.77–0.91	
	model 1	low-rank coal	supercritical CO_2_-water	*D*_A_: 1.795–1.867	0.68–0.72	Song et al.^39^
				*D*_S_: 2.977–2.989	0.71–0.89	
overburden NMR	model 1	2.98 (high rank)	confining pressure: 0–15 MPa	*D*_A_:1.67–1.74	0.47–0.52	Zhang et al.^13^
				*D*_S_: 2.95–2.97	0.91–0.96	
				*D*_T_: 2.51–2.63	0.93–0.94	
	model 1	0.83 (low rank)	confining pressure: 0–12 MPa	*D*_A_: not applicable		Chen et al.^16^
				*D*_S_: 2.78–2.86	0.91–0.94	
				*D*_T_: 2.51–2.63	0.57–0.67	
	model 1	0.62 (low rank)	confining pressure: 0–15 MPa	*D*_S_: 2.932–2.45	None	Cheng et al.^15^
	model 1		confining pressure: 0–12 MPa	*D*_A_: 1.67–1.74		
				*D*_S_: 2.95–2.97		

a *D*_A_, *D*_S_, and *D*_T_ are the fractal dimension values of adsorption pore, percolation pore, and total pore space based on Model 1, respectively, which are dimensionless; *D*_ir_ and *D*_F_ are the fractal dimension values of bound water and movable water distribution based on Model 2, respectively, which are dimensionless.

## 3 Conclusions

This paper presents the results of the LF-NMR technique for characterization of the dynamic changes of pore volume at different diameter scales during pressure drop, desorption–diffusion of adsorbed and free methane, and methane–water interactions. In addition, the calculation principles of NMR single and multiple fractal models are systematically introduced. The significance and applicability of NMR fractal models for characterization in different research contexts are discussed, and the urgent problems to be solved in the application of this technique to CBM production are proposed. The following conclusions are obtained:

(1) Pore–fracture structure evolution. Although the overburden NMR technique has become an emerging method to characterize the stress sensitivity of coal reservoirs, it still has limitations in characterizing the stress sensitivity of unconventional reservoirs, especially for medium- and high-rank coal seams and highly mature shales where fractures are not developed.
(2) Methane desorption transport. Most of the studies in this area focus on the adsorbed/free methane transport under atmospheric pressure (variable pressure) conditions, with emphasis on the interconversion of different phases of methane at different pressure drop rates and the statistical acquisition of the adsorbed/free methane content at different time. The dynamic NMR physical simulation study of reservoir gas–water extraction based on in-situ and actual geological development conditions should become one of the key elements of the subsequent research.
(3) The low-temperature freeze–thaw NMR technique as a mechanistic method has a promising future in both extended NMR simulations and CBM gas injection stimulation. It is still debatable how to study the damage mechanism and pore characterization methods of coal reservoirs simultaneously under low-temperature freeze–thaw using the NMR technology.
(4) The fractal model is one of the main mathematical methods to extend the structure of NMR simulations, while the applicability of NMR fractal models for coal reservoirs has not been reported. In addition, CBM production is a long-term dynamic process and mostly involves calculating the dynamic changes of NMR fractal dimension values for the same coal sample under different confining pressures, desorption pressures, and gas–water action. Compared with static characterization, dynamic characterization of analytical models exhibits more difficulties in terms of applicability. Therefore, the applicability of static and dynamic NMR fractal models needs to be explored further.
